# Modelling inflammatory endothelial dysfunction: a human in vitro platform for translational research

**DOI:** 10.3389/fbioe.2026.1792998

**Published:** 2026-04-09

**Authors:** Maria Cheremkhina, Aaron Babendreyer, Christopher T. Neullens, Susanne Krapp, Alessa Pabst, Kim Ohl, Stephan Ruetten, Andreas Ludwig, Christian G. Cornelissen, Stefan Jockenhoevel, Klaus Tenbrock, Anja Lena Thiebes

**Affiliations:** 1 Department of Biohybrid & Medical Textiles (BioTex), Institute of Applied Medical Engineering, Helmholtz Institute Aachen, RWTH Aachen University, Aachen, Germany; 2 Institute of Molecular Pharmacology, University Hospital RWTH Aachen, Aachen, Germany; 3 Department of Pediatrics, Division of Pediatric Immunology, RWTH Aachen University, Aachen, Germany; 4 Institute of Pathology, Electron Microscopy Facility, RWTH Aachen University Hospital, Aachen, Germany; 5 Department of Pneumology and Internal Intensive Care Medicine, Medical Clinic V, University Hospital RWTH Aachen, Aachen, Germany

**Keywords:** biohybrid medical devices, endothelial dysfunction, in vitro modelling, sepsis, translational device testing

## Abstract

**Introduction:**

Systemic inflammation presents a significant challenge to the long-term function of biohybrid implants. While endothelialisation of biohybrid implants has been shown to improve device hemocompatibility, its feasibility under the influence of patients’ inflammatory status remains largely unexplored. To investigate this, we developed a controlled in vitro model which allows to study endothelial dysfunction under inflammatory stress.

**Methods:**

Endothelial cells were cultured on polydimethylsiloxane under physiological shear stress and exposed to lipopolysaccharide (LPS)-activated peripheral blood mononuclear cells (PBMCs), mimicking systemic inflammation at the blood-material interface. Endothelial morphology and confluence was assessed using immunohistochemistry and scanning electron microscopy. Leukocyte adhesion was evaluated directly as well as indirectly, using flow cytometry to analyse cell adhesion molecules. Quantitative PCR was used for gene expression analysis of inflammatory mediators.

**Results:**

Notably, neither LPS nor PBMCs alone induced endothelial disruption under shear stress, whereas their combination significantly impaired endothelial confluence. Inflammatory activation led to substantial loss of endothelial confluence, increased leukocyte adhesion, and elevated expression of adhesion molecules ICAM-1, VCAM-1, and E-selectin. Gene expression analysis further highlighted the upregulation of inflammatory mediators, such as IL-6, IL-8, IL-10, and MCP-1.

**Discussion:**

This study underscores the challenges of implementing endothelialisation in biohybrid devices, particularly in patients with systemic inflammation. By considering translational hurdles, this work contributes to the development of clinically viable biohybrid constructs and highlights the importance of considering inflammatory dynamics when designing next-generation implants.

## Graphical Abstract

**Figure:**
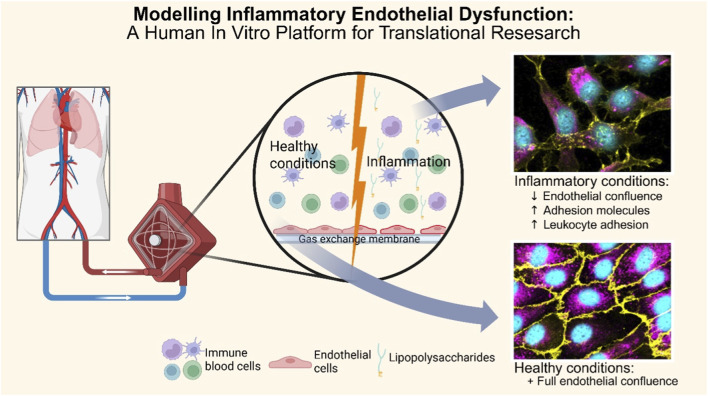
Created in BioRender. Cheremkhina, M. (2026) https://BioRender.com/b9yuiwy.

## 1 Introduction

Biohybrid implants are a promising strategy to improve hemocompatibility of blood-contacting devices. In native vasculature, the endothelium provides a non-thrombogenic layer between the vessel wall and circulating blood and maintains vascular homeostasis by regulating thrombogenesis and inflammatory responses (Zhuang et al., 2021). Artificial implants and devices, such as vascular grafts or oxygenators, are widely used in clinical practice as conventional treatments. However, these devices often have long-term risks due to increased protein adsorption on the device surfaces which initiates thrombus formation and inflammatory responses (Xu et al., 2014; Callaghan et al., 2020). One of the strategies to overcome these challenges is to create a biohybrid device by endothelialisation of the artificial surfaces (Stefopoulos et al., 2016; Zwirner et al., 2018). This approach has been applied to several devices, such as vascular grafts and gas exchange membranes of oxygenators (Melchiorri et al., 2015; Klein et al., 2020). The main challenges of medical device surface endothelialisation include the high wall shear stresses, insufficient cell adhesion to synthetic materials, as well as possible inflammatory activation of the endothelial cells. Robotti et al. showed the ability of endothelial cells to withstand supraphysiological wall shear stresses of up to 60 dyn/cm^2^ without any loss of cell confluency (Robotti et al., 2014). While the cell adhesion to artificial surfaces remains dependent on the surface composition, several surface modifications have been proposed to overcome this issue, including the RGD-modification of polydimethylsiloxane (PDMS) membrane used in the current study (Klein et al., 2020; Akther et al., 2020). General feasibility of endothelialisation has been demonstrated under standard cell culture conditions *in vitro,* for example, for oxygenator membrane (Salehi‐Nik et al., 2017; Alabdullh et al., 2023)*.* Nevertheless, these conditions do not adequately replicate the complex inflammatory state of many patients. For example, high levels of inflammatory cytokines in blood are characteristic of patients undergoing extracorporeal membrane oxygenation (ECMO) treatment, as respiratory failure is often associated with systemic inflammation, whether in acute lung failure or chronic obstructive pulmonary disease (COPD) (Kaku et al., 2020; Wang and Cao, 2021). Moreover, it has been reported that ECMO treatment itself can induce systemic inflammatory response syndrome (SIRS), which leads to increased mortality (Thangappan et al., 2016; Millar et al., 2016).

The behaviour of endothelial cells under inflammatory conditions *in vivo* has been extensively investigated (Joffre et al., 2020; Ince et al., 2016). While endothelial barrier function is maintained in healthy conditions by endothelial cell glycocalyx and intercellular junctions, increased cytokine production during inflammation causes upregulation of adhesion molecules, which in turn allows for leukocyte adhesion and transmigration (Sutton et al., 2014; Hellenthal et al., 2022). This process, while crucial for tissue healing, disrupts intercellular junctions, allowing leukocyte migration to inflamed tissue (Wettschureck et al., 2019). In the context of endothelialised biohybrid implants, this inflammatory response poses a significant challenge: the disruption of intercellular junctions can expose the artificial surface to blood, increasing device thrombogenicity and undermining the biohybrid implant concept.

In the current study, we evaluate the behaviour of endothelial cells on a polydimethylsiloxane (PDMS) gas exchange membrane *in vitro* under inflammatory conditions. Since flow conditions can significantly alter endothelial cell behaviour (Rojas-González et al., 2023), cells are conditioned with relevant wall shear stresses in a microfluidic system. We investigated endothelial cell layer integrity, expression of adhesion molecules and leukocyte adhesion as well as changes in gene expression in standard culture and inflammatory conditions. Our goal is to better predict endothelial cell behaviour in biohybrid devices during future clinical application.

## 2 Materials and methods

### 2.1 Isolation and culture of human umbilical vein endothelial cells

Human umbilical vein endothelial cells (HUVECs) were isolated from umbilical cords according to an established protocol (Menzel et al., 2017). Umbilical cords were provided by the Centralized Biomaterial Bank of the RWTH Aachen University (cBMB) according to its regulations, following RWTH Aachen University, Medical Faculty Ethics Committee (cBMB project number 323) and the Department of Gynaecology and Perinatal Medicine (Univ.-Prof. Dr. Stickeler). The patients’ authorized representative provided informed consent. Culture of HUVECs was performed in endothelial cell growth medium 2 (EGM2, Promocell) with 1% antibiotic-antimycotic solution (ABM, Thermo Fischer) in gelatine-coated (2%, Sigma-Aldrich) tissue-culture flasks (Greiner) in a humidified incubator at 37 °C and 5% CO_2_. Characterization of HUVECs was performed by flow cytometry analysis for CD31 (PECAM-1), CD105, CD144, CD146, and CD90. As shown in the Supplementary Table S1, HUVECs were positive for CD31, CD105, CD144, and CD146, and negative for CD90, which confirmed their endothelial cell characteristics. HUVECs (*n = 3* independent donors) were used in passage 4 for all experiments.

### 2.2 Isolation of peripheral blood mononuclear cells

Peripheral blood mononuclear cells (PBMCs) were isolated from freshly drawn venous blood. Blood was obtained from healthy male donors between 25 and 35 years of age after approval by the local ethics committee of the Medical Faculty of RWTH Aachen University Hospital (EK 22–390) and providing informed consent. Approximately 60 mL of whole blood was used for each isolation. First, whole blood was diluted 1:1 with DPBS. A Pancoll gradient (Pan-Biotech, density: 1.077 g/mL) was then used to separate PBMCs from other blood components. For that, diluted blood was carefully layered onto Pancoll in a ratio of 2:1 (blood:Pancoll), followed by centrifugation for 30 min at 300 g without brakes. The PBMC layer was carefully collected as a layer between plasma and Pancoll (Patrone et al., 2022). Erythrocytes were lysed according to manufacturer´s instructions in erythrocyte lysis buffer (Pan-Biotech) for 5 min at room temperature. PBMCs were used directly after isolation for all experiments.

### 2.3 Static culture of endothelial cells on gas exchange membranes for thrombin generation assay

Polydimethylsiloxane (PDMS) membranes (800 μm, Limitless Shielding) were washed with soap, absolute ethanol (70%, Merck), and ultra-pure water (Sartorius). Circular samples (6 mm diameter) were glued (Elastosil® E50 N Transparent silicon rubber, Wacker Chemie AG) to the bottom of 24-well plates (Greiner). PDMS membranes were functionalised with RGD peptides (a binding motif for cellular integrins) as previously described (Li et al., 2006).

HUVECs were resuspended in medium and seeded with a concentration of 7 × 10^5^ cells/cm^2^. Endothelial cells were incubated for 24 h to allow cell attachment. The membranes were checked for full confluence before performing the thrombin generation assay.

To evaluate the thrombogenicity of the biofunctionalized and endothelialised membranes, a thrombin generation assay (TGA, Haemoscan) was performed. The membranes were carefully washed with DPBS, and the assay was performed according to the manufacturer’s instructions compliant with ISO 10993–4:2017. Briefly, all samples were incubated with human plasma before adding the TGA reagents. Thrombin generation was measured at 1, 2, 3, and 4 min after reagent addition. The optical density was measured at 405 nm and 540 nm with a microplate reader (Infinite® 200Pro, Tecan). Thrombin concentration was calculated from acquired calibration curves. The calculation of maximum thrombin generation was performed according to the manufacturer´s instructions. Briefly, the thrombin generation between 3 and 4 min was normalized to the membrane surface area, as the highest increase in generated thrombin occurred during this time. All samples were evaluated using identically produced membranes to enable relative comparison of thrombin generation between the surfaces.

### 2.4 Dynamic culture of endothelial cells on gas exchange membranes

PDMS membranes were washed as described above, followed by gluing of the membranes to the microfluidic devices (sticky Slides 0.2 Luer, Ibidi) with 250 µm channel height as previously described (Cheremkhina et al., 2023) and coated with RGD. For endothelialisation, HUVECs were resuspended in EGM2 medium with 1% ABM at a concentration of 5 × 10^6^ cells/mL and seeded in the microfluidic channels. Microfluidic devices with seeded cells were incubated statically for at least 2.5 h until full cell attachment while medium exchange was carefully performed every 30 min. Afterwards, three microfluidic devices were connected in series to the bioreactor system as depicted in Supplementary Figure S1. Laminar unidirectional flow of 8.4 mL/min was applied to the cells in microfluidic devices, resulting in a wall shear stress (WSS) of 20 dyn/cm^2^. After 24 h of endothelial cell dynamic culture under standard conditions, the experiment was performed with four different groups: 1) EGM2 served as control, 2) LPS was added to EGM2 in a concentration of 100 ng/mL, 3) PBMCs were diluted in EGM2 with a final concentration of 1.5 × 10^6^ cells/mL, 4) For imitation of inflammatory conditions, LPS-activated PBMCs were added to the culture system at the same concentrations. After 24 h culture under experimental conditions, the culture was terminated by either fixation of the cells with ice-cold methanol (VWR) for immunocytochemistry (ICC) and scanning electron microscopy (SEM), cell detachment with accutase (Innovative Cell Technologies) for flow cytometry, or cell lysis and RNA isolation for quantitative polymerase chain reaction (qPCR) analysis.

### 2.5 Leukocyte adhesion assay

For the leukocyte adhesion assay, PBMCs were stained with 5-chloromethylfluorescein diacetate (CMFDA, 1 μM, Thermo Fisher) for 30 min at 37 °C prior to dynamic culture. A total of 24 × 10^6^ PBMCs in 16 mL EGM2 were used per device, resulting in a final concentration of 1.5 × 10^6^ cells/mL. The dynamic co-culture of PBMCs and endothelial cells was performed under the same conditions as previously mentioned in Section 2.4. Briefly, the co-culture was performed for 24 h under a WSS of 20 dyn/cm^2^. After dynamic culture, microfluidic devices with HUVECs and attached PBMCs were fixed with ice-cold methanol for 10 min at −20 °C, and carefully washed three times with DPBS. Microscopy of the endothelial cells with attached PBMCs was performed (AxioObserver Z1, Carl Zeiss with AxioCam MRm with ZEN blue 3.6 software). Three random fields per sample were analyzed. Endothelialised area of each brightfield image was determined with GNU Image Manipulation Program software (GIMP V2.10.34, The GIMP team) by threshold-based segmentation of confluent endothelial regions. Areas without endothelial coverage were excluded from further analysis. The number of attached PBMCs was calculated with open-source CellProfiler software V4.2.5 (Stirling et al., 2021) using automated object identification. PBMC counts were normalized to the endothelialised surface area within each image.

### 2.6 Immunocytochemical staining

The endothelialised membranes were carefully cut from the microfluidic devices, and ICC was performed with antibodies against CD31 and von Willebrand factor (vWf) and DAPI (Carl Roth) to counterstain nuclei as previously described (Cheremkhina et al., 2023). All used antibodies are listed in Supplementary Table S2. For microscopy, a drop of mounting medium (Ibidi) was placed in a microscopy-suited µ-Slide chambered coverslip (Ibidi), and the sample was placed into the chamber with the cell-side down. Microscopy was performed with an inverted confocal laser scanning microscope (LSM 980 with Airyscan 2, Zeiss). For each condition of each biological donor (n = 3), three independent images were analysed as technical replicates. Analysis was performed using automated segmentation with the Cellpose-SAM framework to identify individual endothelial cells and endothelialized regions (Pachitariu et al., 2025). All segmentation parameters were kept constant across all conditions to ensure comparability between samples: flow_threshold = 0.4, cellprob_threshold = 0.0, and tile_norm_blocksize = 0.

### 2.7 Flow cytometry analysis

After dynamic culture, the cells were washed with DPBS, and treated with accutase for 3 min at 37 °C to ensure gentle cell detachment. After cell collection, live-dead staining (Zombie Aqua, BioLegend) of the cells was performed according to manufacturer’s instructions. After washing, flow cytometry antibody staining was performed according to the manufacturer’s instructions. All used antibodies are listed in Supplementary Table S2. The cells were resuspended in PBS with 1% FBS, and all measurements were performed with FACS Canto cytometer (BD Biosciences). Mean fluorescence intensities (MFI) of the adhesion molecule expression were evaluated and all data were processed using FlowJo software (V10.8.1, FlowJo LLC).

### 2.8 Scanning electron microscopy

SEM analysis was performed by the facility for electron microscopy of the medical faculty of RWTH Aachen University. The methanol-fixed samples were transferred to 3% glutaraldehyde solution (Agar scientific) in 0.1 M sodium phosphate buffer (Merck). Dehydration was performed with ascending ethanol series (30%; 50%; 70%; 90%; 100%; 100%; 100%). Critical point drying in liquid CO_2_ (Polaron, Quorum Technologies Ltd.) was performed before coating of the samples with a 10 nm gold/palladium layer (Sputter Coater EM SCD500, Leica). Scanning electron microscopy was performed in a high vacuum environment at 10 kV voltage (Quattro S, Thermo Fischer Scientific).

### 2.9 Quantitative PCR analysis

RNA was extracted from the cells after dynamic culture by removing the medium from the channels and adding RLT buffer (RNeasy Kit, Qiagen) for cell lysis and consequent RNA isolation. The cells were lyzed without separation of endothelial cells from potentially attached PBMCs in interest of time to process samples for RNA isolation. Collection of RNA from PBMCs was isolated from the cell-medium-suspension, such that no adherent endothelial cells were included. For RNA isolation from PBMCs, medium suspension from dynamic culture was centrifuged at 500 *g* for 5 minutes and the pellet containing PBMCs was lysed with the RLT buffer before RNA isolation according to the manufacturer’s protocol. Finally, RNA was eluted with RNAse-free water. RNA was reverse transcribed using a PrimeScript™ RT Reagent Kit (Takara Bio Europe), and PCR reactions were performed using iTaq Universal SYBR Green Supermix (Bio-Rad), according to the manufacturers’ protocols. The mRNA expression levels of VCAM-1, ICAM-1, E-selectin, IL-8, IL-6, IL-10, TNFα, MCP-1, TM, TPA, vWF, VE-cadherin, CD31, NOS3, EDN1 were measured using quantitative real-time PCR and normalized to the mRNA expression level of different reference genes. Supplementary Table S4 describes all used primers and annealing temperatures. CFX Maestro Software 1.1 (Bio-Rad) was used to determine the most stable reference genes: eukaryotic translation initiation factor 4A2 (EIF4A2) and ribosomal protein L13A (RPL13A). The term reference gene index (ref. gene index) was introduced as the normalization was always performed against these two genes. All PCR reactions were run on a CFX Connect Real-Time PCR Detection System (Bio-Rad) using the following protocol: 40 cycles of 10 s denaturation at 95 °C followed by 10 s annealing and 15 s amplification at 72 °C. The uncorrected RFU values using LinRegPCR version 2020.0 were used to determine the PCR efficiency (Ruijter et al., 2009). The CFX Maestro Software 1.1 (Bio-Rad) was used for the relative quantification, and the evaluation algorithm was based on the ddCt method.

In order to distinguish between the effects coming from endothelial cells and PBMCs, principal component analysis (PCA) was performed based on the qPCR data in Python using the Scikit-learn module (Pedregosa et al., 2011). For that, PBMCs were recovered from the dynamic system and processed separately for gene expression analysis. The standardization of the data was performed using the StandardScaler of Scikit-learn prior to PCA. Plotly Express (Plotly Technologies Inc.) was used for the graphical representation of the results. The samples were coloured depending on the culture conditions. The loadings of the genes were plotted as arrows with the length of the arrow representing the value of the loadings.

### 2.10 Statistical analysis

All experiments were performed with three independent biological replicates (*n* = 3). Each biological replicate consisted of one independent HUVEC donor combined with one independent PBMC donor. Three technical replicates of each biological donor-condition were included in the calculation of endothelialised area and cell count. The results are presented in mean ± standard deviation. The data analysis was performed with Excel 2016 (Microsoft), IBM SPSS Statistics for Windows (Version 29, IBM Corp.) and Prism 9 (9.4.0, Graphpad Software). Statistical evaluation of TGA and leukocyte adhesion assay was performed with Welch´s *t*-test and Mann-Whitney test, respectively. Flow cytometry and qPCR were analysed for the normal distribution using residual plots, Shapiro-Wilk and Kolmogorov-Smirnov tests as diagnostics. In case of normal distribution, ordinary one-way ANOVA with Tukey´s multiple comparisons test was performed for the statistical analysis. A non-parametric Kruskal–Wallis test was performed if no normal distribution was confirmed (Shapiro-Wilk and Kolmogorov-Smirnov tests). Statistical analysis of endothelialised area and cell count was performed using linear mixed models implemented in IBM SPSS Statistics. Culture conditions (endothelial cells without treatment, endothelial cells with LPS, or PBMCs, or combination of PBMCs and LPS) were included as a fixed effect, while donors were included as a random intercept to account for repeated image measurements within donors. Models were fitted using restricted maximum likelihood (REML) estimation with Satterthwaite approximation for degrees of freedom. Pairwise comparisons of estimated means were adjusted using Bonferroni correction for multiple testing. A p-value below 0.05 was considered statistically significant (labelled with *).

## 3 Results

### 3.1 Endothelial cells reduce thrombin generation on gas exchange membranes

**FIGURE 1:**
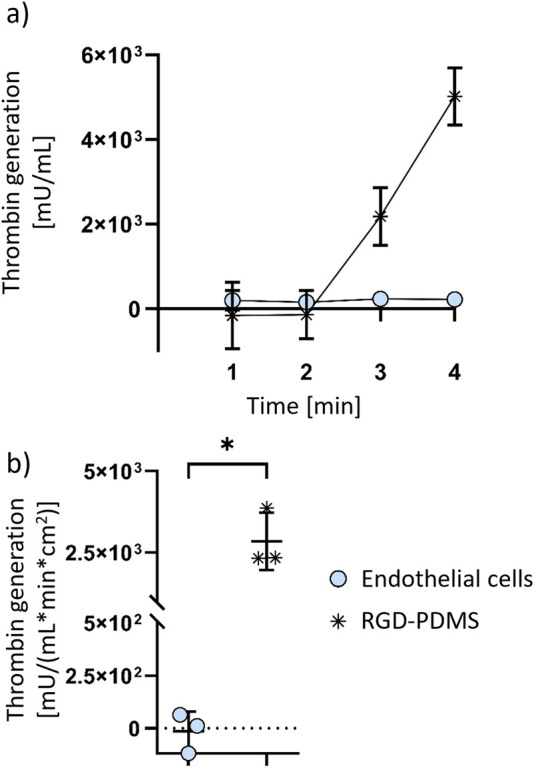
Thrombin generation on endothelialised PDMS membranes in comparison with non-endothelialised RGD-coated PDMS membranes. Thrombin generation over 4 min **(a)**, and maximum thrombin generation during the assay **(b)** are shown. The data is shown as mean ± SD, with symbols in b) representing the individual data points (n = 3 biological HUVEC donors for endothelial cells, or n = 3 technical replicates for RGD-PDMS). P-value below 0.05 was considered statistically significant (labelled with *).

To investigate if endothelial cells improve hemocompatibility of biofunctionalised PDMS gas exchange membranes, thrombin generation is measured (Figure 1). An increase in thrombin generation on RGD-coated PDMS is observed in contrast to endothelialised membranes, on which no thrombin generation occurs during the measurement time (Figure 1a). Thrombin generation is significantly reduced for endothelialised PDMS membranes compared to cell-free membranes, with the maximum thrombin generation of RGD-coated PDMS membrane reaching 2.8 x 10^3^ ± 8.8 x 10^2^ mU/(mL x min x cm^2^), whereas the confluent endothelial cell layer on the PDMS membrane does not induce relevant thrombin generation (Figure 1b).

### 3.2 Inflammatory conditions disrupt the endothelial layer integrity

**FIGURE 2:**
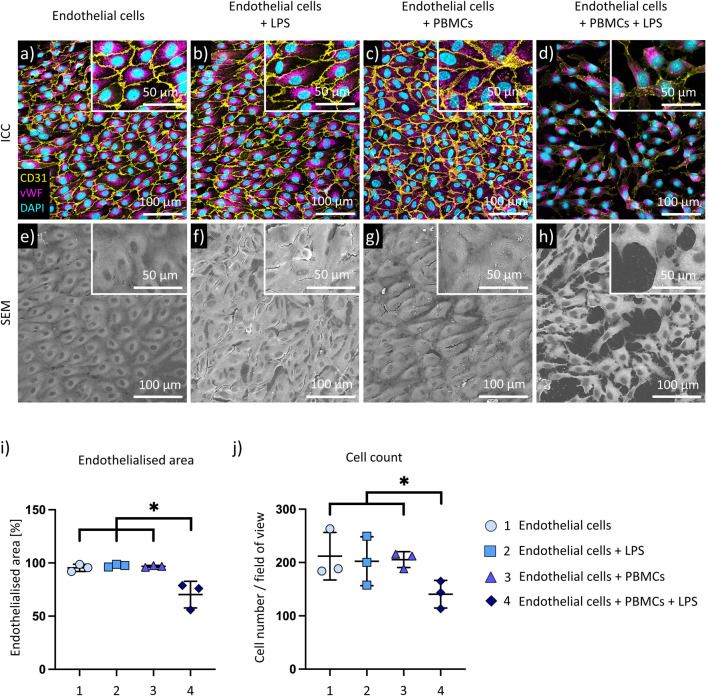
Evaluation of endothelial cell morphology. Immunocytochemical staining of endothelial cells against CD31 (yellow), vWF (purple), and DAPI (blue)-stained cell nuclei **(a–d)**; scanning electron microscopy of endothelial cell layer after dynamic culture **(e–h)**. Representative images (n = 3 biological replicates) of following conditions are shown: endothelial cells (medium control) **(a,e)**; endothelial cells with LPS-treatment **(b,f)**; endothelial cells cultured with PBMCs **(c,g)**; endothelial cells cultured with LPS-treated PBMCs **(d,h)**. Quantitative analysis of endothelialised surface coverage **(i)** and endothelial cell number **(j)** performed using automated segmentation with the Cellpose-SAM framework. Data is presented as mean ± SD, and data is considered statistically significant with p-value below 0.05.

Maintenance of a confluent endothelial cell layer is essential for successful application of biohybrid devices. ICC staining and SEM are performed to evaluate the ability of endothelial cells to withstand WSS and preserve cell layer confluence during dynamic culture under inflammatory conditions (Figures 2d,h). Endothelial cells dynamically cultured with either only LPS or PBMCs or in standard cell culture medium are used as controls (Figures 2a–c,e–g).

Cell-cell adhesions are shown by CD31 staining (yellow), while vWF is found in the cytoplasm (pink).

Culture of endothelial cells with either LPS or PBMCs does not change their morphology or expression pattern of CD31 (Figures 2e–g,a–c, respectively). In PBMC-only and LPS-only groups (Figures 2f,g), local points with intercellular gaps were observed in SEM. As these gaps are observed in IHC staining (Figures 2b,c), this is likely to be a technical artefact which resulted from dehydration and critical point drying during SEM sample preparation. In contrast, a loss of cell confluence is observed upon culture of endothelial cells under inflammatory conditions. The endothelial cell layer appears disrupted (Figures 2d,h) and a substantial reduction of CD31-cell-cell adhesions is confirmed by antibody staining (Figure 2d). vWF expression does not seem to be altered by culture under inflammatory conditions (Figure 2d) in comparison to the controls (Figures 2a–c). Quantitative analysis of endothelial cell coverage (Figure 2I) and cell number (Figure 2j) confirmed the morphological observations shown in Figures 2a–h, demonstrating significant decline in endothelial cell confluence and cell number upon culture under inflammatory conditions.

### 3.3 Inflammatory conditions increase leukocyte adhesion

**FIGURE 3:**
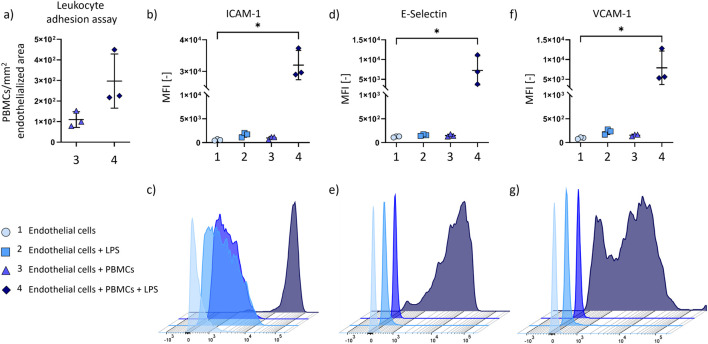
Leukocyte adhesion on endothelial cells (n = 3 biological replicates) during dynamic culture under inflammatory conditions. Leukocyte adhesion assay **(a)** comparing PBMC-adhesion to endothelial cells with and without LPS-activation (n = 3 biological replicates). Flow cytometry **(b–g)** of endothelial cells for following cell adhesion molecules: **(b,c)** Intercellular adhesion molecule-1 (ICAM-1); **(d,e)** E-Selectin; **(f,g)** Vascular cell adhesion molecule 1 (VCAM-1). Mean fluorescence intensity (MFI) of adhesion molecules expression calculated from n = 3 independent donors are shown **(b,d,f)**, as well as representative histograms of one donor **(c,e,g)**. P-value below 0.05 was considered statistically significant (labelled with *).

Leukocyte adhesion to endothelial cells is a key characteristic of inflammatory processes *in vivo*. In this work, it is directly and indirectly evaluated by measuring leukocyte adhesion (Figure 3a) and expression of adhesion molecules ICAM-1, E-Selectin, and VCAM-1 (Figures 3b–g) via flow cytometry, respectively.

Adhesion of PBMCs is increased on endothelial cells under LPS-treatment (Figure 3a). In detail, 110 ± 31 PBMCs/mm^2^ adhere to endothelial cells during LPS-free culture, and 297 ± 107 PBMCs/mm^2^ to endothelial cells with LPS-treatment. Representative pictures of endothelial cells treated with stained PBMCs, with and without LPS activation, are shown in Supplementary Figure S3. Flow cytometry reveals a significant increase in ICAM-1, E-Selectin, and VCAM-1 expression for culture of endothelial cells with LPS-activated PBMCs in comparison to the medium control (Figures 3b–g, see Supplementary Table S3 for all MFI data).

### 3.4 Inflammatory conditions induce the expression of pro-inflammatory proteins in endothelial cells

**FIGURE 4:**
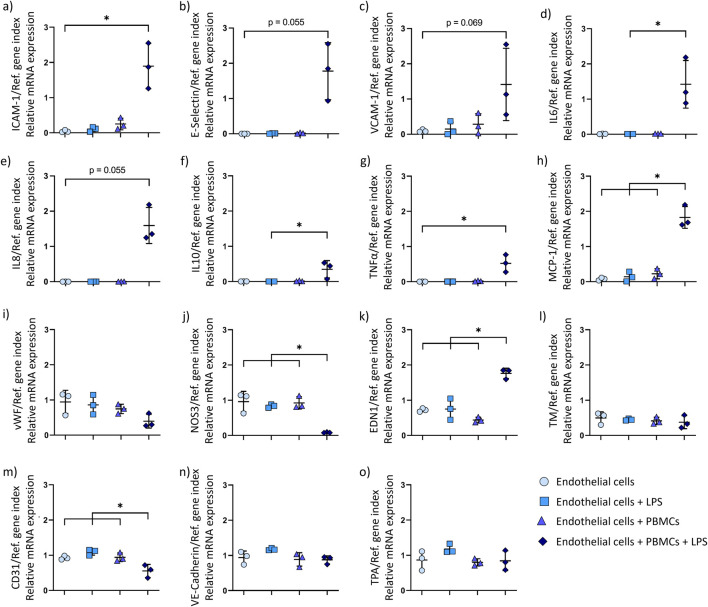
Relative mRNA expression in endothelial cells (n = 3 biological replicates) after dynamic culture under inflammatory conditions (endothelial cells + PBMCs + LPS), with following groups as controls: endothelial cells, endothelial cells + LPS, endothelial cells + PBMCs. Endothelial cells were analysed for relative mRNA expression of **(a)** Intercellular adhesion molecule-1 (ICAM-1); **(b)** E-Selectin; **(c)** Vascular cell adhesion molecule 1 (VCAM-1); **(d)** Interleukin 6 (IL6); **(e)** Interleukin 8 (IL8); **(f)** Interleukin 10 (IL10); **(g)** Tumor necrosis factor alpha (TNFα); **(h)** Monocyte chemoattractant protein-1 (MCP-1); **(i)** Von Willebrand factor (vWF); **(j)** Nitric oxide synthase 3 (NOS3); **(k)** Endothelin 1 (EDN1); **(l)** Thrombomodulin (TM); **(m)** Platelet and endothelial cell adhesion molecule 1 (PECAM-1, CD31); **(n)** Vascular endothelial cadherin (VE-Cadherin); **(o)** Tissue plasminogen activator (TPA) in relation to a reference gene index consisting of EIF4A2 and RPL13A. Data are shown as mean ± SD with symbols representing the individual data points. P-value below 0.05 was considered statistically significant (labelled with *).

Endothelial cell behaviour during dynamic culture under inflammatory conditions is evaluated by quantitative mRNA analysis. The results are shown in Figure 4. Expression of the following genes is analysed to investigate inflammatory processes: ICAM-1, E-Selectin, VCAM-1, IL6, IL8 (gene CXCL8), IL10, TNFα, and MCP-1 (gene CCL2) (Figures 4a–h). The mRNA expression of endothelial adhesion molecules ICAM-1, VCAM-1, and E-Selectin is increased upon culture under inflammatory conditions, with a significant result for ICAM-1 expression compared to the control. Similarly, a higher expression of the pro-inflammatory genes IL6 and IL8, as well as the anti-inflammatory gene IL10 is observed in inflammatory conditions, along with a significant increase in TNFα and MPC-1 expression. No significant differences are observed in the expression of the following genes involved in thrombogenicity regulation in endothelial cells: vWF, TM (gene THBD), and TPA (gene PLAT) (Figures 4i,l,o). NOS3 gene expression is significantly reduced, while its antagonist, EDN1, shows a significant increase in endothelial cells cultured under inflammatory conditions (Figures 4j,k). No change in VE-Cadherin (gene CDH5) expression is observed (Figure 4n). Expression of CD31 (gene PECAM-1), is significantly decreased in inflammatory conditions (Figure 4m).

**FIGURE 5:**
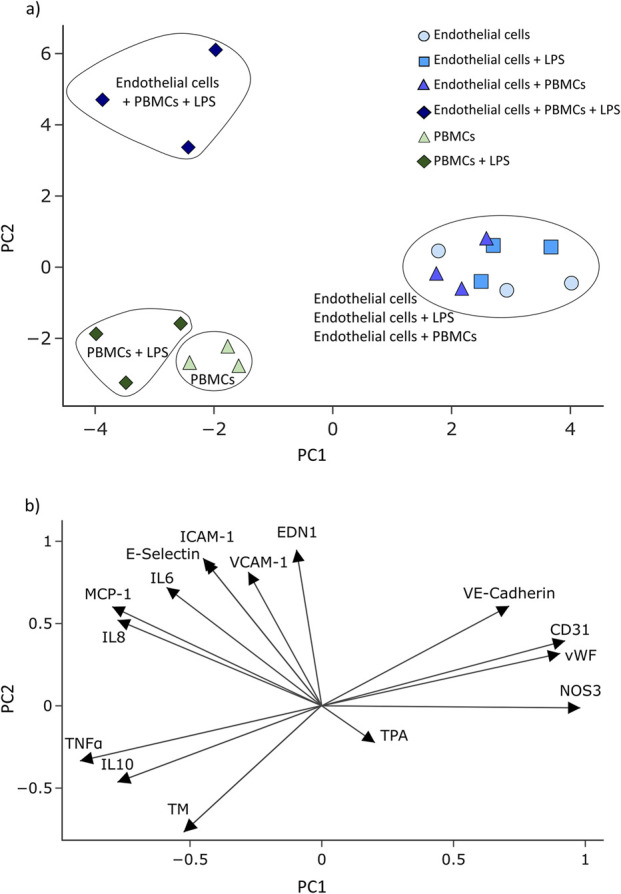
Principal component analysis of mRNA expression data. Results are presented as scatter plots where each symbol represents one sample. PBMC and PBMC + LPS samples correspond to immune cells recovered from the respective endothelial cell co-culture. Samples are coloured for the different culture conditions **(a)**. The loadings of investigated genes are plotted as arrows **(b)**.

A multidimensional PCA was performed to further evaluate qPCR data (Figure 5). PCA showed a clear separation of endothelial cells dynamically cultured under inflammatory conditions from all control groups (Figure 5a). Furthermore, clear separation of PBMCs from all endothelial cell groups was observed, with an additional separation between the PBMCs and LPS-activated PBMCs (Figure 5a). Three groups of genes can be identified considering the loadings of the genes (Figure 5b). While the mRNA expression of ICAM-1, VCAM-1, E-Selectin, IL6, IL8, MCP-1, and EDN1 affect the grouping of endothelial cells cultured under inflammatory conditions, mRNA expression of VE-Cadherin, CD31, vWF, and NOS3 genes appears to be relevant for endothelial cells cultured under all other conditions. PBMCs (with and without LPS) strongly express TNFα and IL10 mRNA (Figure 5b).

## 4 Discussion

In the current study, we highlight the importance of replicating physiological conditions closely *in vitro* to advance the translational potential of biohybrid medical devices. Specifically, endothelialisation of gas exchange membranes has been proposed in several studies as a possible solution to overcome hemocompatibility issues of ECMO devices, mainly by proof-of-concept studies as well as methodologies for endothelialisation and the evaluation of the ability of endothelial cells to maintain confluence and functionality during long-term dynamic culture under non-inflammatory conditions (Klein et al., 2020; Alabdullh et al., 2023; Pflaum et al., 2021). A WSS of 20 dyn/cm^2^ was applied in the current study to better approximate hemodynamic conditions expected in endothelialised biohybrid blood-contacting devices. Evaluating endothelial behaviour under these conditions provides a device-relevant model for assessing endothelial responses in biohybrid devices.

We measured thrombin generation to evaluate if the thrombogenicity of PDMS membranes improves by endothelialisation. The result supports the general hypothesis that endothelialisation of artificial membranes reduces membrane thrombogenicity and thus improves the devices’ hemocompatibility. We assume that the thrombogenicity of endothelialised membrane remains significantly lower than that of uncoated membranes, as long as the endothelial cell layer is fully confluent and does not expose the membrane surface underneath it (Siedlecki, 2024).

An inflammatory status has been reported in many patients whose condition could be treated with a biohybrid device such as endothelialzed ECMO, or a vascular graft (Millar et al., 2016; Sapienza et al., 2019; Sterpetti et al., 2020). Systemic inflammatory response is characterized by highly elevated pro- and anti-inflammatory cytokine levels (Datzmann and Traeger, 2018). Endothelial cells respond to inflammation with endothelial activation which eventually results in increased leukocyte adhesion, decreased barrier function, vasoconstriction, and vascular leakage and changes in thromboresistance (Sutton et al., 2014). This inflammation-induced vascular dysfunction is reversible with appropriate treatment *in vivo* (Wu et al., 2020). To the authors’ knowledge, no investigation of endothelial cells on artificial membranes has been performed under inflammatory conditions. Thus, we used a dynamic microfluidic culture system that allows for dynamic culture of endothelial cells on gas exchange membranes under inflammatory conditions imitated by the addition of LPS-activated PBMCs. Essential differences have been previously observed in the endothelial cell response to cytokine induction in static versus dynamic culture conditions, indicating the importance of evaluating the endothelial cell response to inflammation under dynamic conditions (Shaw et al., 2001). Under static conditions, immune cells are known to sediment on the endothelial cell layer, resulting in non-physiological cell-cell interactions and increased local cell contact. In contrast, laminar flow provides continuous movement of PBMCs across the endothelial cell surface, providing a more realistic simulation of immune-endothelial interactions *in vivo* (Lohasz et al., 2025).

Evaluation of endothelial cell layer after inflammatory treatment showed a loss of endothelial cell layer confluence. CD31 expression significantly decreased in endothelial cells under inflammatory conditions on mRNA and protein level. CD31 regulates vascular permeability, as well as inhibits the activation of circulating thrombocytes and leukocytes (Lertkiatmongkol et al., 2016). A combination of cytokines TNFα and interferon-γ (IFNγ) has been reported to decrease CD31 expression (Woodfin et al., 2007). Consistent with these findings, we observed upregulation of TNFα gene expression in endothelial cells after inflammatory treatment. Notably, endothelial cells and PBMCs can express TNFα (Lee and Nevo, 2021; Alinejad et al., 2022). According to the PCA of qPCR results, the TNFα gene expression primarily occurs in LPS-activated PBMCs. Thus, we assume that the reduction in CD31 expression in endothelial cells occurs due to increased TNFα expression in LPS-activated PBMCs.

*In vivo*, leukocyte adhesion is an essential part of the defence mechanisms during inflammation. After activation, leukocytes tend to accumulate around the site of inflammation (van de Vijver et al., 2013). Leukocytes start a rolling movement along the endothelial cells, attaching to the endothelial cell adhesion molecule E-Selectin (Rosen and Bertozzi, 1996). ICAM-1 and VCAM-1, as ligands for lymphocyte function adhesion molecule-1 (LFA-1) and very late activation antigen-4 (VLA-4), respectively, allow leukocytes to firmly adhere to the endothelial cells (Lawson et al., 2009). Following, leukocytes transmigrate through the endothelial cell layer towards the inflamed tissue. Although a necessary and reversible process during healthy vascular physiology, leukocyte transmigration can result in vascular leak, expanding permeability of endothelial cell layer in pathological conditions (Weis, 2008). Consistent with the *in vivo* reaction of endothelial cells to inflammation, we observed an increase in endothelial cell adhesion molecules E-Selectin, ICAM-1 and VCAM-1 on protein expression level. These results confirm endothelial cell activation resulting in increased leukocyte adhesion during culture under inflammatory conditions. We assume that the lack of underlying tissue further promotes endothelial cell detachment and restricts the endothelial cells from restoring the confluence.

Because cyto- and chemokines are an important mechanism for intercellular communication, we evaluated the gene expression of several pro- and anti-inflammatory factors. Upregulation of IL6 and MCP-1 gene expression in endothelial cells under inflammatory conditions confirms cell activation. This is associated with vascular leakage under septic conditions, which is consistent with our results (Kang and Kishimoto, 2021). Additionally, MCP-1 is responsible for attracting leukocytes to the site of injury (Singh et al., 2021). In contrast, IL10 acts as an anti-inflammatory cytokine. IL10 has been shown to inhibit pro-inflammatory cytokine production, i.e., TNFα and IFNγ, and support wound healing and tissue repair (Moore et al., 2001; Short et al., 2022). In our study, a significant increase in IL10 gene expression was detected under inflammatory conditions. PCA revealed that IL10, similar to TNFα, is mostly expressed by PBMCs and not endothelial cells in the microfluidic system. Thus, we assume that the observed upregulation of IL10 comes as an effect from the RNA being isolated from endothelial cells with the attached PBMCs. Altogether, IL10 expressed by PBMCs might be a potential treatment target to decrease pro-inflammatory cytokine expression and promote endothelial cell layer recovery.

NOS3 is responsible for nitric oxide (NO) production, and thereby reduces thrombocyte reactivity and thrombogenicity of endothelial cells (Oliveira-Paula et al., 2016). The reduction in NOS3 gene expression in endothelial cells under inflammatory conditions could lead to a decrease in NO production. In a biohybrid device setup, this in turn could lead to an increased thrombosis risk in patients with inflammation. NOS3 protects against systemic inflammation by reducing ICAM-1 and IL6 expression, which makes it an interesting potential therapeutic target for inflammation treatment (Bougaki et al., 2010). During inflammation, the disruption of vascular homeostasis leads to upregulation of EDN1, which acts as an antagonist to NOS3 (Gupta, 2022). This has been confirmed by our investigation of EDN1 gene expression. LPS-induced sepsis is associated with increased EDN1 expression (Kowalczyk et al., 2015). In endothelial cells, EDN1 binds to endothelin receptor type B (ETB) that leads to an increased NOS3 activation, and in turn caused NO-induced vasodilation (Enevoldsen et al., 2020). In general, reduced expression of NOS3 and increased expression of EDN1 in endothelial cells during inflammation points to endothelial cell dysfunction due to KLF2 expression destabilisation (Dabravolski et al., 2022).

The study represents the first investigation of endothelialised membranes for potential application in a biohybrid device model under inflammatory conditions. We deem this essential before proceeding with (pre-) clinical studies to prove the feasibility of biohybrid devices. Our study emphasizes the importance of (biohybrid) device testing in conditions similar to later application in patients. A condition such as inflammation can drastically influence the potential device performance, as shown in this study.

A limitation of the present study is the use of HUVECs as cell line for endothelialisation. Although HUVECs represent a well-established and standardized endothelial cell model for *in vitro* studies, they are mostly not a feasible option for clinical application. Autologous patient-derived endothelial cells, e.g., from fat tissue, endothelial progenitor cells, or human induced pluripotent stem cell derived endothelial cells (iPSC-ECs) could be a feasible cell source. In particular, it has been shown that iPSC-ECs exhibit a generally reduced immunogenic and inflammatory response compared to primary endothelial cells like HUVECs (Jia et al., 2024). Further studies on biohybrid device endothelialisation should investigate the behaviour of alternative clinical need-suited endothelial cells under inflammatory and shear stress conditions to further assess the translational applicability of the model.

In this study, the 24 h evaluation time point was selected to compare different experimental conditions. Future studies could integrate live imaging to narrow down the exact period of onset of confluence loss and understand the chronological sequence of events in inflammatory endothelial responses.

In the setup used for this study, the reactions of endothelial cells to complex inflammatory stimuli provided by the combination of PBMCs and LPS very closely mimic the reactions observed *in vivo*. The setup is highly flexible: the culture substrate can easily be exchanged, wall shear stresses can be varied, and immune blood cell or LPS concentration can be modified as well as the used cell types changed, e.g., by adding granulocytes or thrombocytes. Its use extends to a versatile disease model not only for the study of endothelial behaviour in sepsis but for the complex interactions between endothelial cells and the immune system. Future studies could incorporate analyses following the withdrawal of inflammatory stimuli to investigate possible endothelial cell recovery. Moreover, the presented model can serve as a platform for testing anti-inflammatory drugs, to restore the barrier integrity.

The flexibility of our design could also allow for changes in the flow type. In the current study, we used well-controlled laminar flow conditions to allow standardized evaluation of endothelial cell responses to inflammatory stimuli under reproducible mechanical stimulation. However, the hemodynamic environment in, e.g., cardiovascular biohybrid devices can be more complex. *In vivo*, endothelial cells might be exposed to turbulent flow patterns, as well as circumferential stretch. Although this goes beyond the scope of this study, these cell culture conditions could further challenge endothelial stability on artificial surfaces. Still, while this study investigates inflammatory endothelial dysfunction under defined laminar flow, further studies should include different flow patterns to investigate endothelial cell performance in more complex hemodynamic environments.

The presented setup allowed us to investigate endothelial cell behaviour on artificial membranes under inflammatory conditions. Exposure to LPS-activated PBMCs causes loss of endothelial cell layer confluence, as well as increased leukocyte adhesion, accompanied by upregulation of inflammation-associated genes in endothelial cells. The observed loss of endothelial cell layer integrity puts a big question mark on the concept of biohybrid device endothelialisation under inflammatory conditions. An endothelial layer failing already after 24 h would expose the underlying artificial surface, triggering thrombus formation and negating the intended benefits. This work highlights the need for careful consideration of patient inflammatory status in the design and evaluation of biohybrid devices. Future studies should incorporate such translational insights to refine device strategies, ensuring their efficacy and safety in clinical application.

## Data Availability

The raw data supporting the conclusions of this article will be made available by the authors, without undue reservation.

## Glossary

ABM -: antibiotic-antimycotic solution
CD31 -: Cluster of differentiation 31
CMFDA -: 5-Chloromethylfluorescein diacetate
COPD -: Chronic obstructive pulmonary disease
DAPI -: 4′,6-Diamidino-2-phenylindole
DPBS -: Dulbecco’s phosphate buffered saline
ECMO -: Extracorporeal membrane oxygenation
EIF4A2 -: Eukaryotic translation initiation factor 4A2
EDN1 -: Endothelin 1
EGM2 -: Endothelial growth medium 2
ETB -: Endothelin receptor type B
FACS -: Fluorescence-activated cell sorting
ICAM-1 -: Intercellular adhesion molecule 1
IFNγ -: Interferon gamma
IL-6 -: Interleukin 6
IL-8 -: Interleukin 8
IL-10 -: Interleukin 10
KLF2 -: Krüppel-like factor 2
LFA-1 -: Lymphocyte function-associated antigen 1
LPS -: Lipopolysaccharide
MCP-1 -: Monocyte chemoattractant protein 1
MFI -: Mean fluorescence intensity
NO -: Nitric oxide
NOS3 -: Nitric oxide synthase 3
PBMCs -: Peripheral blood mononuclear cells
PCA -: Principal component analysis
PECAM-1 -: Platelet endothelial cell adhesion molecule 1
PDMS -: Polydimethylsiloxane
qPCR -: Quantitative polymerase chain reaction
RGD -: Arginine-Glycine-Aspartate
RNA -: Ribonucleic acid
RFU -: Relative fluorescence units
RPPL13A-: Ribosomal protein L13A
SEM -: Scanning electron microscopy
SIRS -: Systemic inflammatory response syndrome
TGA -: Thrombin generation assay
TNFα -: Tumor necrosis factor alpha
TM -: Thrombomodulin
TPA -: Tissue plasminogen activator
VE-Cadherin -: Vascular endothelial cadherin
vWf -: von Willebrand factor
VCAM-1 -: Vascular cell adhesion molecule 1
VLA-4 -: Very late antigen 4
WSS -: Wall shear stress

## References

[B1] Akther F. Yakob S. B. Nguyen N.-T. Ta H. T. (2020). Surface modification techniques for endothelial cell seeding in PDMS microfluidic devices. Biosensors 10 (11), 182. 10.3390/bios10110182 33228050 PMC7699314

[B2] Alabdullh H. A. Pflaum M. Mälzer M. Kipp M. Naghilouy-Hidaji H. Adam D. (2023). Biohybrid lung development: towards complete endothelialization of an assembled extracorporeal membrane oxygenator. Bioengineering 10 (1), 72. 10.3390/bioengineering10010072 36671644 PMC9854558

[B3] Alinejad S. Khademvatan S. Amani S. Asadi N. Tappeh K. H. Yousefi E. (2022). The effect of curcumin on the expression of INFγ, TNF-α, and iNOS genes in PBMCs infected with Leishmania major [MRHO/IR/75/ER]. Infect. Disorders-Drug Targets Formerly Curr. Drug Targets-Infectious Disord. 22 (6), 83–89. 10.2174/1871526522666220404083220 35379161

[B4] Bougaki M. Searles R. J. Kida K. De Yu J. Buys E. S. Ichinose F. (2010). Nos3 protects against systemic inflammation and myocardial dysfunction in murine polymicrobial sepsis. Shock (Augusta, Ga.) 34 (3), 281–290. 10.1097/SHK.0b013e3181cdc327 19997049 PMC3774000

[B5] Callaghan S. Cai T. McCafferty C. Van Den Helm S. Horton S. MacLaren G. (2020). Adsorption of blood components to extracorporeal membrane oxygenation (ECMO) surfaces in humans: a systematic review. J. Clin. Med. 9 (10), 3272. 10.3390/jcm9103272 33053879 PMC7601136

[B6] Cheremkhina M. Klein S. Babendreyer A. Ludwig A. Schmitz-Rode T. Jockenhoevel S. (2023). Influence of aerosolization on endothelial cells for efficient cell deposition in biohybrid and regenerative applications. Micromachines 14 (3), 575. 10.3390/mi14030575 36984982 PMC10053765

[B8] Dabravolski S. A. Sukhorukov V. N. Kalmykov V. A. Grechko A. V. Shakhpazyan N. K. Orekhov A. N. (2022). The role of KLF2 in the regulation of atherosclerosis development and potential use of KLF2-targeted therapy. Biomedicines 10 (2), 254. 10.3390/biomedicines10020254 35203463 PMC8869605

[B9] Datzmann T. Traeger K. (2018). Extracorporeal membrane oxygenation and cytokine adsorption. J. Thoracic Disease 10 (Suppl. 5), S653–S660. 10.21037/jtd.2017.10.128 PMC5911550 29732183

[B10] Enevoldsen F. C. Sahana J. Wehland M. Grimm D. Infanger M. Krüger M. (2020). Endothelin receptor antagonists: status quo and future perspectives for targeted therapy. J. Clin. Med. 9 (3), 824. 10.3390/jcm9030824 32197449 PMC7141375

[B11] Gupta A. (2022). “An overview of gene variants of Endothelin-1: a critical regulator of endothelial dysfunction,” in Endothelial Dysfunction-A novel paradigm.

[B12] Hellenthal K. E. Brabenec L. Wagner N.-M. (2022). Regulation and dysregulation of endothelial permeability during systemic inflammation. Cells 11 (12), 1935. 10.3390/cells11121935 35741064 PMC9221661

[B13] Ince C. Mayeux P. R. Nguyen T. Gomez H. Kellum J. A. Ospina-Tascón G. A. (2016). The endothelium in sepsis. Shock 45 (3), 259–270. 10.1097/SHK.0000000000000473 26871664 PMC5281063

[B14] Jia H. Moore M. Wadhwa M. Burns C. (2024). Human iPSC–derived endothelial cells exhibit reduced immunogenicity in comparison with human primary endothelial cells. Stem Cells Int. 2024 (1), 6153235. 10.1155/sci/6153235 39687754 PMC11649354

[B15] Joffre J. Hellman J. Ince C. Ait-Oufella H. (2020). Endothelial responses in sepsis. Am. Journal Respiratory Critical Care Medicine 202 (3), 361–370. 10.1164/rccm.201910-1911TR 32101446

[B16] Kaku S. Nguyen C. D. Htet N. N. Tutera D. Barr J. Paintal H. S. (2020). Acute respiratory distress syndrome: etiology, pathogenesis, and summary on management. J. Intensive Care Med. 35 (8), 723–737. 10.1177/0885066619855021 31208266

[B17] Kang S. Kishimoto T. (2021). Interplay between interleukin-6 signaling and the vascular endothelium in cytokine storms. Exp. & Molecular Medicine 53 (7), 1116–1123. 10.1038/s12276-021-00649-0 34253862 PMC8273570

[B18] Klein S. Hesselmann F. Djeljadini S. Berger T. Thiebes A. L. Schmitz-Rode T. (2020). EndOxy: dynamic long-term evaluation of endothelialized gas exchange membranes for a biohybrid lung. Ann. Biomed. Eng. 48 (2), 747–756. 10.1007/s10439-019-02401-2 31754901 PMC6949203

[B19] Kowalczyk A. Kleniewska P. Kolodziejczyk M. Skibska B. Goraca A. (2015). The role of endothelin-1 and endothelin receptor antagonists in inflammatory response and sepsis. Archivum Immunol. Ther. Exp. 63, 41–52. 10.1007/s00005-014-0310-1 25288367 PMC4289534

[B20] Lawson C. Wolf S. (2009). ICAM-1 signaling in endothelial cells. Pharmacol. Reports 61 (1), 22–32. 10.1016/s1734-1140(09)70004-0 19307690

[B21] Lee D. K. Nevo O. (2021). Tumor necrosis factor alpha expression is increased in maternal microvascular endothelial cells in preeclampsia. Hypertens. Pregnancy 40 (3), 193–201. 10.1080/10641955.2021.1921794 33979559

[B22] Lertkiatmongkol P. Liao D. Mei H. Hu Y. Newman P. J. (2016). Endothelial functions of PECAM-1 (CD31). Curr. Opinion Hematology 23 (3), 253–259. 10.1097/MOH.0000000000000239 27055047 PMC4986701

[B23] Li B. Chen J. Wang J. H. (2006). RGD peptide-conjugated poly(dimethylsiloxane) promotes adhesion, proliferation, and collagen secretion of human fibroblasts. J. Biomed. Mater. Res. A 79 (4), 989–998. 10.1002/jbm.a.30847 16948145

[B24] Lohasz C. Häfeli T. Hasanova D. Hölting L. Rudnik M. Ligeon L.-A. (2025). A microfluidic platform for the co-culturing of microtissues with continuously recirculating suspension cells. Microsystems & Nanoeng. 11 (1), 184. 10.1038/s41378-025-01028-9 41073406 PMC12514218

[B25] Melchiorri A. Hibino N. Yi T. Lee Y. Sugiura T. Tara S. (2015). Contrasting biofunctionalization strategies for the enhanced endothelialization of biodegradable vascular grafts. Biomacromolecules 16 (2), 437–446. 10.1021/bm501853s 25545620 PMC4325601

[B26] Menzel S. Finocchiaro N. Donay C. Thiebes A. L. Hesselmann F. Arens J. (2017). Towards a biohybrid lung: endothelial cells promote oxygen transfer through gas permeable membranes. Biomed. Res. Int. 2017, 5258196. 10.1155/2017/5258196 28913354 PMC5587952

[B27] Millar J. E. Fanning J. P. McDonald C. I. McAuley D. F. Fraser J. F. (2016). The inflammatory response to extracorporeal membrane oxygenation (ECMO): a review of the pathophysiology. Crit. Care 20 (1), 1–10. 10.1186/s13054-016-1570-4 27890016 PMC5125043

[B28] Moore K. W. de Waal Malefyt R. Coffman R. L. O'Garra A. (2001). Interleukin-10 and the interleukin-10 receptor. Annu. Review Immunology 19 (1), 683–765. 10.1146/annurev.immunol.19.1.683 11244051

[B29] Oliveira-Paula G. H. Lacchini R. Tanus-Santos J. E. (2016). Endothelial nitric oxide synthase: from biochemistry and gene structure to clinical implications of NOS3 polymorphisms. Gene 575 (2), 584–599. 10.1016/j.gene.2015.09.061 26428312 PMC6728140

[B30] Pachitariu M. Rariden M. Stringer C. (2025). Cellpose-SAM: superhuman generalization for cellular segmentation. BioRxiv. 2025:2025.04.28.651001. 10.1101/2025.04.28.651001

[B31] Patrone D. Alessio N. Antonucci N. Brigida A. L. Peluso G. Galderisi U. (2022). Optimization of peripheral blood mononuclear cell extraction from small volume of blood samples: potential implications for children-related diseases. Methods Protoc. 5 (2), 20. 10.3390/mps5020020 35314657 PMC8938807

[B32] Pedregosa F. Varoquaux G. Gramfort A. Michel V. Thirion B. Grisel O. (2011). Scikit-learn: machine learning in python. The J. Machine Learn. Research 12, 2825–2830.

[B33] Pflaum M. Jurmann S. Katsirntaki K. Malzer M. Haverich A. Wiegmann B. (2021). Towards biohybrid lung development-fibronectin-coating bestows hemocompatibility of gas exchange hollow fiber membranes by improving flow-resistant endothelialization. Membr. (Basel) 12 (1), 35. 10.3390/membranes12010035 35054561 PMC8779364

[B34] Robotti F. Franco D. Bänninger L. Wyler J. Starck C. T. Falk V. (2014). The influence of surface micro-structure on endothelialization under supraphysiological wall shear stress. Biomaterials 35 (30), 8479–8486. 10.1016/j.biomaterials.2014.06.046 25017097

[B35] Rojas-González D. M. Babendreyer A. Ludwig A. Mela P. (2023). Analysis of flow-induced transcriptional response and cell alignment of different sources of endothelial cells used in vascular tissue engineering. Sci. Rep. 13 (1), 14384. 10.1038/s41598-023-41247-6 37658092 PMC10474151

[B36] Rosen S. D. Bertozzi C. R. (1996). Leukocyte adhesion: two selectins converge on sulphate. Curr. Biol. 6 (3), 261–264. 10.1016/s0960-9822(02)00473-6 8805242

[B37] Ruijter J. Ramakers C. Hoogaars W. Karlen Y. Bakker O. Van den Hoff M. (2009). Amplification efficiency: linking baseline and bias in the analysis of quantitative PCR data. Nucleic Acids Research 37 (6), e45. 10.1093/nar/gkp045 19237396 PMC2665230

[B38] Salehi‐Nik N. Banikarimi S. P. Amoabediny G. Pouran B. Shokrgozar M. A. Zandieh‐Doulabi B. (2017). Flow preconditioning of endothelial cells on collagen‐immobilized silicone fibers enhances cell retention and antithrombotic function. Artif. Organs 41 (6), 556–567. 10.1111/aor.12759 27418522

[B39] Sapienza P. Mingoli A. Borrelli V. Grande R. Sterpetti A. V. Biacchi D. (2019). Different inflammatory cytokines release after open and endovascular reconstructions influences wound healing. Int. Wound J. 16 (4), 1034–1044. 10.1111/iwj.13154 31158921 PMC7949274

[B40] Shaw S. K. Perkins B. N. Lim Y.-C. Liu Y. Nusrat A. Schnell F. J. (2001). Reduced expression of junctional adhesion molecule and platelet/endothelial cell adhesion molecule-1 (CD31) at human vascular endothelial junctions by cytokines tumor necrosis factor-α plus interferon-γ does not reduce leukocyte transmigration under flow. Am. Journal Pathology 159 (6), 2281–2291. 10.1016/s0002-9440(10)63078-7 11733377 PMC1850595

[B41] Short W. D. Steen E. Kaul A. Wang X. Olutoye O. O. Vangapandu H. V. (2022). IL‐10 promotes endothelial progenitor cell infiltration and wound healing via STAT3. FASEB J. 36 (7), e22298. 10.1096/fj.201901024RR 35670763 PMC9796147

[B42] Siedlecki C. (2024). Hemocompatibility of biomaterials for clinical applications: blood-biomaterials interactions. Sawston, United Kingdom: Woodhead Publishing (Elsevier). 10.1016/C2014-0-04140-8

[B43] Singh S. Anshita D. Ravichandiran V. (2021). MCP-1: function, regulation, and involvement in disease. Int. Immunopharmacology 101, 107598. 10.1016/j.intimp.2021.107598 34233864 PMC8135227

[B44] Stefopoulos G. Robotti F. Falk V. Poulikakos D. Ferrari A. (2016). Endothelialization of rationally microtextured surfaces with minimal cell seeding under flow. Small 12 (30), 4113–4126. 10.1002/smll.201503959 27346806

[B45] Sterpetti A. V. Sapienza P. Borrelli V. Di Marzo L. (2020). Inflammatory cytokines and experimental arterial and vein grafts. JTCVS Techniques 1, 48–50. 10.1016/j.xjtc.2020.01.008 34317710 PMC8289074

[B46] Stirling D. R. Swain-Bowden M. J. Lucas A. M. Carpenter A. E. Cimini B. A. Goodman A. (2021). CellProfiler 4: improvements in speed, utility and usability. BMC Bioinformatics 22, 1–11. 10.1186/s12859-021-04344-9 34507520 PMC8431850

[B47] Sutton N. R. Baek A. Pinsky D. J. (2014). “Endothelial cells and inflammation,” in Encyclopedia of medical immunology: autoimmune diseases. Editors Mackay I. R. Rose N. R. Diamond B. Davidson A. (New York, NY: Springer New York), 367–381.

[B48] Thangappan K. Cavarocchi N. C. Baram M. Thoma B. Hirose H. (2016). Systemic inflammatory response syndrome (SIRS) after extracorporeal membrane oxygenation (ECMO): incidence, risks and survivals. Heart & Lung 45 (5), 449–453. 10.1016/j.hrtlng.2016.06.004 27425197

[B49] van de Vijver E. van den Berg T. K. Kuijpers T. W. (2013). Leukocyte adhesion deficiencies. Hematology/Oncology Clin. 27 (1), 101–116. 10.1016/j.hoc.2012.10.001 23351991

[B50] Wang Y. Cao P. (2021). Extracorporeal membrane oxygenation (ECMO) for acute exacerbations of chronic obstructive pulmonary disease: care modalities, experience, and precautions. Am. J. Transl. Res. 13 (5), 4922–4927. 34150076 PMC8205735

[B51] Weis S. M. (2008). Evaluating vascular leak *in vivo* . Methods Enzymology 444, 99–114. 10.1016/S0076-6879(08)02805-X 19007662

[B52] Wettschureck N. Strilic B. Offermanns S. (2019). Passing the vascular barrier: endothelial signaling processes controlling extravasation. Physiol. Reviews 99 (3), 1467–1525. 10.1152/physrev.00037.2018 31140373

[B53] Woodfin A. Voisin M.-B. Nourshargh S. (2007). PECAM-1: a multi-functional molecule in inflammation and vascular biology. Arteriosclerosis, Thrombosis, Vascular Biology 27 (12), 2514–2523. 10.1161/ATVBAHA.107.151456 17872453

[B54] Wu J. Deng Z. Sun M. Zhang W. Yang Y. Zeng Z. (2020). Polydatin protects against lipopolysaccharide-induced endothelial barrier disruption via SIRT3 activation. Lab. Investig. 100 (4), 643–656. 10.1038/s41374-019-0332-8 31641228

[B55] Xu L.-C. Bauer J. W. Siedlecki C. A. (2014). Proteins, platelets, and blood coagulation at biomaterial interfaces. Colloids Surfaces B Biointerfaces 124, 49–68. 10.1016/j.colsurfb.2014.09.040 25448722 PMC5001692

[B56] Zhuang Y. Zhang C. Cheng M. Huang J. Liu Q. Yuan G. (2021). Challenges and strategies for *in situ* endothelialization and long-term lumen patency of vascular grafts. Bioact. Mater. 6 (6), 1791–1809. 10.1016/j.bioactmat.2020.11.028 33336112 PMC7721596

[B57] Zwirner U. Höffler K. Pflaum M. Korossis S. Haverich A. Wiegmann B. (2018). Identifying an optimal seeding protocol and endothelial cell substrate for biohybrid lung development. J. Of Tissue Eng. And Regen. Med. 12 (12), 2319–2330. 10.1002/term.2764 30362254

