# Supplementary material for: Modelling inflammatory endothelial dysfunction: a human in vitro platform for translational research
Source: Front Bioeng Biotechnol. 2026 Apr 9;14:1792998. doi: 10.3389/fbioe.2026.1792998 (PMC13102812; doi:10.3389/fbioe.2026.1792998)
Supplement: Supplementary file 1 [file DataSheet1.pdf]

Supplementary Material

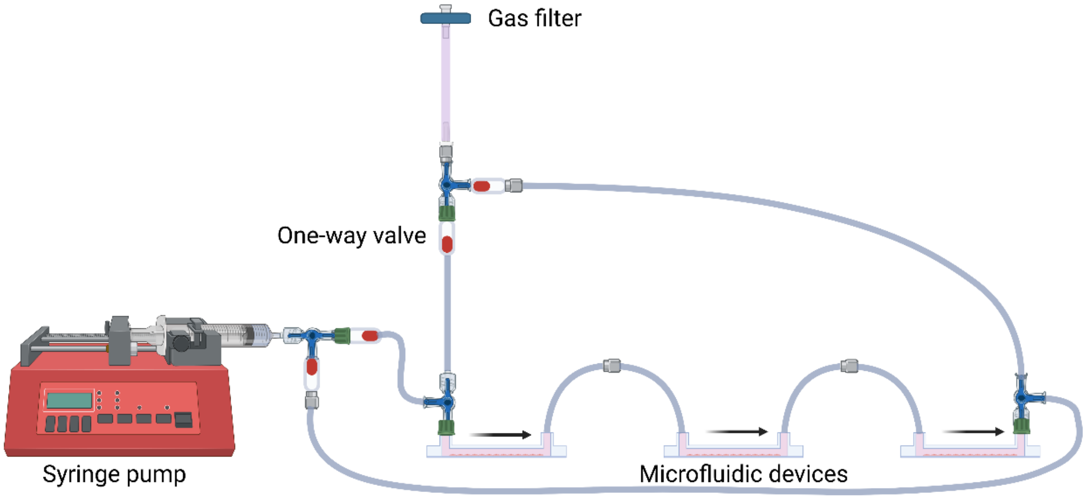

Figure S1. Bioreactor system with syringe pump and connected microfluidic devices. The bioreactor system was assembled using a syringe pump (LA-120, Landgraf Laborsysteme GmbH), syringe (B.Braun), gas filter (Merck), three-way valves (B.Braun), one-way valves (CODAN Medizinische Geräte GmbH) and Heidelberger extensions (B.Braun). Including one-way valves allowed for one-directional flow inside the microfluidic chambers during the syringe's pushing and pulling phase. The bioreactor system was assembled and cultured with EGM2 medium with 1% ABM at 37 °C and 5% CO2 24 h prior to connection of microfluidic devices for system equilibration to reduce the amount of air bubbles in the tubing. Created in BioRender. Cheremkhina, M. (2026) https://BioRender.com/9q2cngd.

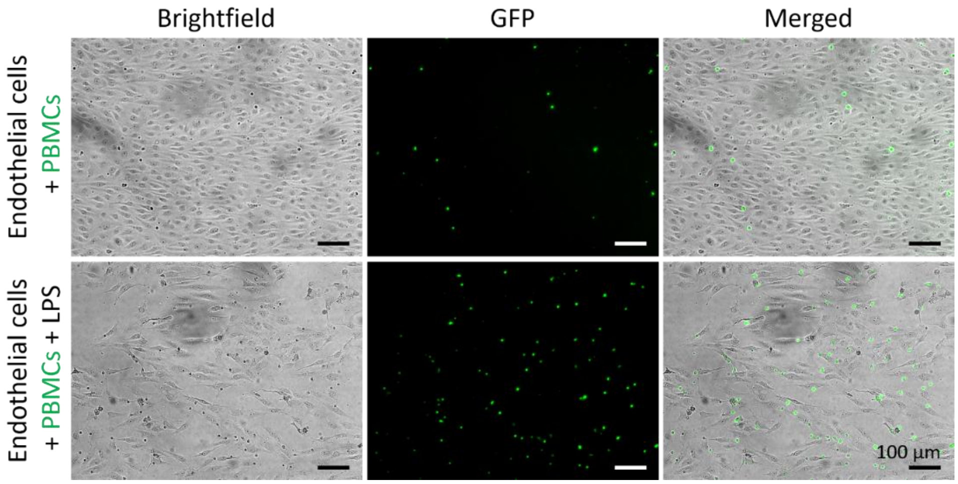

Figure S2. Representative images of leukocyte adhesion assay. PBMCs (green) dynamically cultivated on endothelial cells (brightfield) with or without LPS-activation. Scale bar: 100 µm.

Table S1. Percentage of positive HUVECs (n=4) stained for four endothelial cell-characteristic markers (CD31, CD105, CD144, and CD146) and one mesenchymal stromal cell-characteristic marker (CD90).

Antibody	*Percentage of positive cells Mean ± SD [%]*
CD31 (PECAM-1)	100 ± 0
CD105 (Endoglin)	99.98 ± 0.05
CD144 (VE-cadherin)	98.03 ± 1.26
CD146 (MCAM)	91.53 ± 15.63
CD90	3.08 ± 1.84

Table S2. Antibodies used for immunocytochemistry (ICC) and flow cytometry (FC)

**Analysis method**	**Primary antibody**			**Secondary antibody**			
	Antigen	*Conc*	*Manufacturer information*	*Reactivity*	*Conjugate*	*Conc.*	*Manufacturer information*
**ICC**	CD31, mouse	1:100	P8590, Sigma Aldrich	goat anti-mouse	Alexa Fluor 594	1:400	A11005, Thermo Fischer Scientific
**ICC**	vWf, rabbit	1:100	A0082, Dako	goat anti-rabbit	Alexa Fluor 488	1:400	A11008, Thermo Fischer Scientific
**FC**	CD54 (ICAM-1)	0.5:100	555510, BD Biosciences	rat anti-mouse	BD Horizon V450	0.5:100	562107, BD Biosciences
	**Coupled antibodies**						
	Antigen	*Conjugate*	*Concentration*	*Manufacturer information*			
**FC**	CD106 (VCAM-1)	PE	15:100	555647, BD Biosciences			
**FC**	CD62E (E-Selectin)	FITC	15:100	BBA21, R&D Systems			

Table S3. Mean Fluorescence Intensity (MFI) of ICAM-1, E-Selectin, and VCAM-1 expression measured by flow cytometry of endothelial cells (n=3) under the following conditions: no treatment, treatment with LPS, treatment with PBMCs, and treatment with LPS-activated PBMCs.

Adhesion molecule	*Condition*	*MFI Mean ± SD [-]*
ICAM-1	Endothelial cells	569.7 ± 122
	Endothelial cells + LPS	1607.3 ± 396
	Endothelial cells + PBMCs	972 ± 249.7
	Endothelial cells + PBMCs + LPS	31994.7 ± 3795.8
E-Selectin	Endothelial cells	122 ± 9.9
	Endothelial cells + LPS	155.3 ± 14.7
	Endothelial cells + PBMCs	148 ± 21.2
	Endothelial cells + PBMCs + LPS	7256.3 ± 3024.3
VCAM-1	Endothelial cells	94 ± 14.3
	Endothelial cells + LPS	226.3 ± 44.2
	Endothelial cells + PBMCs	154 ± 16.3
	Endothelial cells + PBMCs + LPS	7918.7 ± 3465.4

Table S4. Primers used for qPCR and their annealing temperatures.

Gene	*Sequence*	*Annealing temperature*
ICAM-1	forward: GGAGCCCGCTGAGGTCACGA<br>reverse: CGCTGGCAGGACAAAGGTCTGG	66 °C
E-Selectin	forward: TTGCCCTATGCTACACAG<br>reverse: TTGAGTCCACTGAAGCCA	56 °C
VCAM-1	forward: GCAAGTCTACATATCACCC<br>reverse: AATCTTCCATCCTCATAGCA	57 °C
IL6	forward: GTGTGAAAGCAGCAAAGAG<br>reverse: AAGTCTCCTCATTGAATCCA	57 °C
IL8 (gene CXCL8)	forward: GACATACTCCAAACCTTTCC<br>reverse: AACTTCTCCACAACCCTC	60 °C
IL10	forward: GCTGTCATCGATTTCTTC<br>reverse: GTCAAACTCACTCATGGC	60 °C
TNFα	forward: TGAGCACTGAAAGCATGATCC<br>reverse: CGAGAAGATGATCTGACTGCC	60 °C
MCP-1 (gene CCL2)	forward: ATGAAAGTCTCTGCCGCC<br>reverse: CTTCTTTGGGACACTTGCT	57 °C
vWF	forward: TACCACAACCACCTGCCT<br>reverse: GTAAGTGAAGCCCGACCGA	57 °C
TM (gene THBD)	forward: AGAGAAGAGACAAACACCT<br>reverse: TCCACAAGACCAGTAGAG	57 °C
TPA (gene PLAT)	forward: TGCTACTTTGGGAATGGG<br>reverse: GTTCTGTGCTGTGTAAACCT	57 °C
NOS3	forward: CGAGTGAACGCGACAATCCT<br>reverse: GCTGCAAAGCTCTCTCCATTC	60 °C
EDN1	forward: CCTAAGACAAACCAGGTCGG<br>reverse: CTTTGCCAGTCAGGAACCA	60 °C
VE-Cadherin (gene CDH5)	forward: TCAAGCGTGAGTCCGCAAGAA<br>reverse: AATGACAGCAGTGAGGTGGT	60 °C
CD31 (gene PECAM-1)	forward: CAGCCAACTTCACCATCC<br>reverse: GAGAGCATTTCACATACGAC	57 °C
EIF4A2	forward: CCAAAAGGTAATTCTGGCACTTG<br>reverse: CGGGTGTACCAACAACAATATGT	60 °C
RPL13A	forward: GCCCTACGACAAGAAAAGCG<br>reverse: TACTTCCAGCCAACCTCGTGA	60 °C
